# Indoor Simulated Training Environment for Brain-Controlled Wheelchair Based on Steady-State Visual Evoked Potentials

**DOI:** 10.3389/fnbot.2019.00101

**Published:** 2020-01-08

**Authors:** Ming Liu, Kangning Wang, Xiaogang Chen, Jing Zhao, Yuanyuan Chen, Huiquan Wang, Jinhai Wang, Shengpu Xu

**Affiliations:** ^1^Institute of Biomedical Engineering, Chinese Academy of Medical Sciences and Peking Union Medical College, Tianjin, China; ^2^School of Electronics and Information Engineering, Tianjin Polytechnic University, Tianjin, China; ^3^Institute of Traditional Chinese Medicine, Tianjin University of Traditional Chinese Medicine, Tianjin, China; ^4^School of Microelectronics, Tianjin University, Tianjin, China

**Keywords:** simulated environment, brain-controlled wheelchair, indoor training, steady-state visual evoked potentials, path recommendation

## Abstract

Brain-controlled wheelchair (BCW) has the potential to improve the quality of life for people with motor disabilities. A lot of training is necessary for users to learn and improve BCW control ability and the performances of BCW control are crucial for patients in daily use. In consideration of safety and efficiency, an indoor simulated training environment is built up in this paper to improve the performance of BCW control. The indoor simulated environment mainly realizes BCW implementation, simulated training scenario setup, path planning and recommendation, simulated operation, and scoring. And the BCW is based on steady-state visual evoked potentials (SSVEP) and the filter bank canonical correlation analysis (FBCCA) is used to analyze the electroencephalography (EEG). Five tasks include individual accuracy, simple linear path, obstacles avoidance, comprehensive steering scenarios, and evaluation task are designed, 10 healthy subjects were recruited and carried out the 7-days training experiment to assess the performance of the training environment. Scoring and command-consuming are conducted to evaluate the improvement before and after training. The results indicate that the average accuracy is 93.55% and improves from 91.05% in the first stage to 96.05% in the second stage (*p* = 0.001). Meanwhile, the average score increases from 79.88 in the first session to 96.66 in the last session and tend to be stable (*p* < 0.001). The average number of commands and collisions to complete the tasks decreases significantly with or without the approximate shortest path (*p* < 0.001). These results show that the performance of subjects in BCW control achieves improvement and verify the feasibility and effectiveness of the proposed simulated training environment.

## Introduction

A brain-computer interface (BCI) provides a new communication and control channel between the human brain and the external world without depending on peripheral nerves and muscles, which helps users interact with an external environment directly (Wolpaw et al., 2002; Naseer and Hong, 2015). There are various non-invasive methods for obtaining brain signals in BCI systems such as electroencephalography (EEG) (Abiri et al., 2019), functional near-infrared spectroscopy (Khan and Hong, 2017; Hong et al., 2018), functional magnetic resonance imaging (Sitaram et al., 2008), and magnetoencephalography (Mellinger et al., 2007). Specifically, brain-controlled wheelchair (BCW) is a particular device based on BCI, which is able to provide assistance and potentially improve the quality of life for people who have no ability to control a wheelchair by conventional interfaces due to some diseases, such as motor neuron diseases, total paralysis, stroke, etc. (Rebsamen et al., 2010). Technically, the signals obtained from spontaneous or evoked brain activities are used to generate and send commands operating the wheelchair. The common types include event-related desynchronization (ERD)/event-related synchronization (ERS)-based BCW (Tanaka et al., 2005; Wang and Bezerianos, 2017), P300-based BCW (Iturrate et al., 2009; Rebsamen et al., 2010; Yu et al., 2017), steady-state somatosensory evoked potentials (SSSEP)-based BCW (Kim et al., 2018), steady-state visual evoked potentials (SSVEP)-based BCW (Diez et al., 2013; Müller et al., 2013) and hybrid BCW (Long et al., 2012; Cao et al., 2014; Li et al., 2014).

To our knowledge, the first report of BCW was published by Tanaka and coworkers, in which the motor imagery (MI) tasks were adopted to control a wheelchair and the accuracy is close to 80% (Tanaka et al., 2005). Rebsamen et al. (2006) combined P300 and path guidance to steer the wheelchair in an office-like environment without complex sensors. Müller et al. (2015) used low and high frequency SSVEP to control the BCW and the corresponding average accuracies of disabled subjects are 54% and 51%, but the high frequency stimuli are more comfortable. In recent years, systems with shared control and different levels of artificial intelligence were introduced into BCW to improve the driving safety (Rebsamen et al., 2007; Galán et al., 2008; Satti et al., 2011; Tang et al., 2018). Some simulated systems were also built to test the feasibility of the related designs. According to reports by Leeb et al. (2007), a tetraplegic is able to control movements of the wheelchair through EEG in a virtual environment. Gentiletti et al. (2009) designed a simulation platform based on P300 and verified the practicability through wheelchair control, and Herweg et al. (2016) reported a virtual environment for wheelchair control based on P300 as well. Through audio-cued MI-based BCI, Francisco et al. (2013) achieved wheelchair control in virtual and real environments. Besides, Wang and coworkers also proposed multiple patterns of MI to implement the movement control of the virtual automatic car (Wang et al., 2019). In terms of the simulated systems based on SSVEP-based BCI or hybrid BCI, Bi et al. (2014) applied SSVEP-based BCI to control a simulated vehicle and Li et al. (2018) combined hybrid BCI with computer vision to build a simulated driving system.

However, for the development of BCW, the practical application is still a critical problem (Yu et al., 2017). It is difficult and, to some extent, dangerous for patients to control BCW in complex situations, especially for naive users (Bi et al., 2013; Fernández-Rodríguez et al., 2016). In addition, the proficiency and efficiency of BCW control are crucial for patients in daily use (Fernández-Rodríguez et al., 2016). Therefore, it is desirable to exploit efficient systems to improve the BCW control performance of users. Recently, many studies have shown that training is one of the effective ways to improve the performance of subjects in BCI (Herweg et al., 2016; Wan et al., 2016; Yao et al., 2019). Hence, we hold the opinion that training can be capable of improving the ability to control the BCW of users, which has rarely been adopted in previous reports. Meanwhile, it is useful for the calibration of parameters (e.g., threshold values) and performance test of BCW (Gentiletti et al., 2009; Li et al., 2018). Furthermore, rehabilitation experts believe that the motivation of patients in training plays an important role in the recovery of motor control (Kaufman and Becker, 1986; Griffiths and Hughes, 1993; Maclean et al., 2000). Nowadays, training in the simulated environment is a better choice because of safety, convenience, and low consumption (Bi et al., 2013). It has been proved that the disabled are able to perform motor learning and task training in the virtual environment and transfer the learned skills to real world performance (Kenyon and Afenya, 1995; Rose et al., 2000). In some cases, the skills can even be extended to other untrained tasks and have good effects (Todorov et al., 1997; Holden, 2005). At the same time, simulated training in a familiar indoor environment (e.g., home or hospital) is more helpful, because users (patients) spend most of their time in home or hospital.

Nevertheless, in the previous researches, few indoor simulated environments were proposed for BCW control training according to different situations and levels of difficulty. In addition, effective training experiments and evaluation methods are also necessary. Taking into account these factors, an indoor simulated training environment based on SSVEP-based BCI is presented in this paper. This training environment integrated training and control in the processes from BCW implementation, training scenario setting, path planning and recommendation, simulated control training, and finally to scoring. And a 7-days simulated training experiment with four training sessions and five tasks was conducted to evaluate the feasibility. The reasons for choosing SSVEP-based BCI are as follows. From the aspect of practical application, SSVEP-based BCI is more suitable for BCW to issue control commands due to its fast command issuing and more stable performance (Bi et al., 2013, 2014). In most situations, view switching between the stimuli and the environment needs to be trained repeatedly as well. Secondly, SSVEP-based BCI generally requires only short-term calibration and training processes of the systems, so the results of performance improvement obtained by the 7-days training experiment are convincing.

The hypothesis in this paper is that training in our simulated training environment can lead to an increase of the accuracy and scores, and eventually the performance enhancement of the users who never used BCW before. In this study, 10 healthy subjects were recruited and asked to carry out training experiments to assess the feasibility and performance of the indoor simulated training environment. This paper is organized as follows. In see section “Materials and Methods,” the methods and materials of the system are introduced. In section Results, experimental results are presented. See section “Discussion” provides discussions. At last, some conclusions are given in see section “Conclusion.”

## Materials and Methods

**FIGURE 1:**
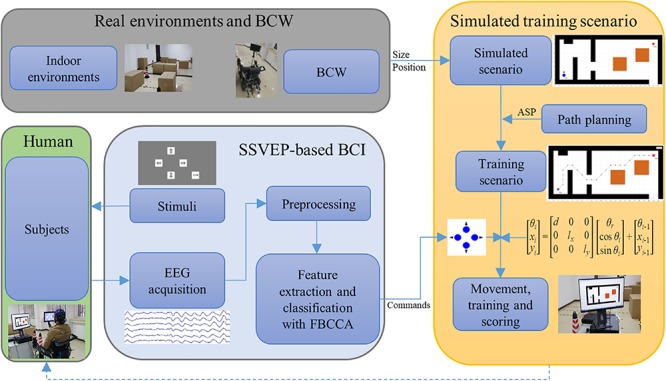
The schematic of the indoor simulated training environment.

The schematic of the indoor simulated training environment is as shown in Figure 1. The main functions of the environment include SSVEP-based BCI input, training scenario setup, path planning and recommendation, simulated operation, and scoring. The SSVEP-based BCI uses a sampled sinusoidal stimulation method to evoke SSVEP (Nikolay et al., 2013) and utilize a filter bank canonical correlation analysis (FBCCA) method to improve the detection accuracies of SSVEP by incorporating fundamental and harmonic frequency components (Chen et al., 2015). The simulated scenario is modeled from a real indoor environment with BCW and objects (obstacles) placed in it. The approximate shortest path (ASP) is provided by using the A-star algorithm owing to its simplicity and efficiency (Koenig and Likhachev, 2005; Le et al., 2018). Subjects are able to steer according to the scenario with or without path planning.

### System Hardware

**FIGURE 2:**
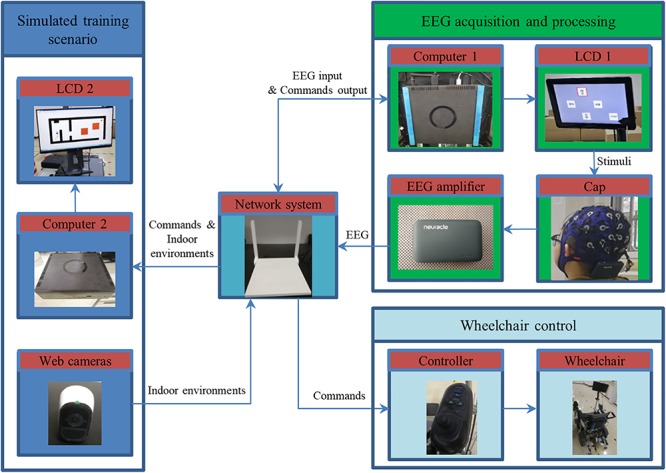
The hardware block diagram of the system.

The relevant hardware of the system is as shown in Figure 2, which mainly includes three parts: EEG acquisition and processing, wheelchair control, and simulated training scenario. And data exchange between the various parts is implemented by the network system (router). The associated hardware mainly consists of a wheelchair, two liquid-crystal display (LCD) screens, a minicomputer, a wireless EEG amplifier, three web cameras, and a server computer.

The minicomputer (Computer 1) includes Intel core CPU (i5-7500 T, 64-bit), 32 GB RAM, and 8 GB video card (Nvidia GTX 1070). The stimulus programs and data analysis are running in Computer 1 using MATLAB (MathWorks, Inc.). The stimuli are presented on a 12.1-inch LCD screen (LCD 1) with a resolution of 1280 × 800 pixels and a 60 Hz refresh rate. EEG data are recorded by a wireless EEG amplifier (Neuracle, Inc.) with an EEG cap. The ordinary powered wheelchair (DYW-459-46A6, 0.55 m width and 1.10 m length) is used and a wireless module is added, so it can be controlled by both the joystick and Wi-Fi pattern. In addition, a server computer (Computer 2) is used to run the training program with a 26-inch LCD screen (LCD 2) to display the training scenario and three web cameras are connected to the server to obtain the situation of the room. The parameters (e.g., size and position) of BCW and obstacles in the simulated scenario are set according to the images captured by the cameras.

### Stimulation and Data Acquisition

**FIGURE 3:**
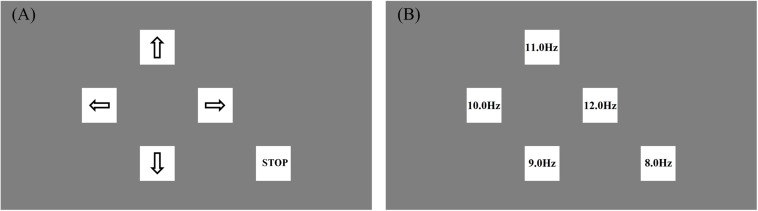
Distribution **(A)** and frequency values **(B)** of the stimulus targets.

The functional modules of our SSVEP-based BCW mainly include stimuli, EEG acquisition, feature extraction and classification, and control. The screen luminance of the stimulus sequence *s*(*f*_*k*_,*∅*,*i*_*s*_) is modulated by the sampled sinusoidal stimulation method as the following equation (Wang et al., 2010; Nikolay et al., 2013):

(1) $$s\,\left(f_{k},\emptyset ,i_{s}\right)=\frac{1}{2}\,\left\{1+\text{sin}\,\left[2\,\pi \,f_{k}\,\left(\frac{i_{s}}{f_{r}}\right)+\emptyset \right]\right\}$$

where *f*_*k*_ is the stimulus frequency; *∅* represents the initial phase; *i*_*s*_ is the frame index of the stimulus (*i*_*s*_ = 0, 1, 2, …); *f*_*r*_ is the refresh rate of the screen and set at 60 Hz. In order to get a stronger response and avoid harmonic interference, the range of *f*_*k*_ is commonly set from 8 Hz to less than 16 Hz (Pastor et al., 2003; Chen et al., 2014). The number of stimulus targets is determined by *f*_*k*_ and *∅*. The basic control intentions (commands) of BCW are turn-right, move-forward, turn-left, move-backward and stop, so *k* is set at an integer from 1 to 5. The corresponding frequencies *f*_*k*_ is set at 12, 11, 10, 9, and 8 Hz, and *∅* is set at 0. Figure 3 shows the distribution and frequency values of the stimulus targets.

Six Ag/AgCl electrodes (O1, Oz, O2, PO3, POz, and PO4 of the international 10–20 system) over the occipital and parietal areas are used to acquire SSVEP with the ground electrode at the midpoint of FPz and Fz and the reference electrode at the vertex (Cz). The signals of six channels are acquired at a sampled rate of 250 Hz when the electrode impedances are below 10 kΩ, the recording data ***X***_EEG_ is processed by the band-pass filtered at 1–100 Hz and the notch filter at 50 Hz. Event trigger signal occupies another channel to synchronize the EEG data and the stimulus event.

### Data Analysis

In consideration of the accuracy and efficiency of feature extraction and classification, FBCCA is adopted to achieve frequency detection in this study (Chen et al., 2015). FBCCA is an extended method to improve the accuracy of canonical correlation analysis (CCA) in the frequency detection of SSVEP. It decomposes the full frequency range of EEG into sub-bands and calculates the correlation coefficients between each sub-bands and the reference signal, respectively. The maximum weighted sum of the correlation coefficients is used to determine the classified results of SSVEP.

In CCA, the reference signal ***Y***_*fk*_ corresponding to the stimulus frequency *f*_*k*_ is as following (Lin et al., 2007):

(2) $${\text{Y}}_{f_{k}}=[\text{sin}\left(2\pi f_{k}t\right),\text{cos }\left(2\pi f_{k}t\right),\ldots ,\text{sin }\left(2\pi N_{h}f_{k}t\right),\text{cos}\left(2\pi N_{h}f_{k}t\right)]{}^{\text{T}}$$

where *N*_*h*_ is the number of harmonics; *t* is equal from $\frac{1}{f_{s}}$ to $\frac{N_{s}}{f_{s}}$, in which *f*_*s*_ is the sampling rate and *N*_*s*_ is the number of sampling points. The linear combination of two variables ***x*** and ***y*** are set as ***x*** = ***X***^T^***W***_**X**_ and ***y*** = ***Y***^T^***W***_**Y**_. And the maximum correlation coefficient of two variables *ρ*(***x***, ***y***) is calculated via:

(3) $$\rho \,\left(x,y\right)=\underset{W_{X},W_{Y}}{\text{max}}\frac{E\,\left({\text{W}}_{X}^{\text{T}}\,{\text{XY}}^{\text{T}}\,{\text{W}}_{Y}\right)}{\sqrt{E\,\left({\text{W}}_{X}^{\text{T}}\,{\text{XX}}^{\text{T}}\,{\text{W}}_{X}\right)\,E\,\left({\text{W}}_{Y}^{\text{T}}\,{\text{YY}}^{\text{T}}\,{\text{W}}_{Y}\right)}}$$

In FBCCA, we first acquire the maximum correlation coefficients between each sub-bands and reference signals by CCA and then calculate the weighted sum in the full frequency range. The weighted sum ${\tilde{\rho }}_{k}$ of the maximum correlation coefficients represents as follows:

(4) $${\tilde{\rho }}_{k}=\sum_{n=1}^{N}\left(n^{-a}+b\right)(\rho_{k}^{n}({\text{X}}_{{\mathrm{SB}}_{n}}^{\text{T}}{\text{W}}_{X}\left({\text{X}}_{{\mathrm{SB}}_{n}},{\text{Y}}_{f_{k}}\right),{\text{Y}}_{f_{k}}^{\text{T}}{\text{W}}_{Y}\left({\text{X}}_{{\mathrm{SB}}_{n}},{\text{Y}}_{f_{k}}\right))){}^{2}$$

where $\rho_{k}^{n}$() is the maximum correlation coefficients and calculated by Eq. (3), *n* (*n* = 1:*N*) and *k* (*k* = 1:5) are the indexes and represent the sequence number of sub-bands and stimulus frequencies, respectively. ***X*_SBn_** represents the signal of the *n*-th sub-band obtained by filter bank analysis from ***X***_EEG_. Namely, serial band-pass filters are used to extract sub-bands. Since the useful harmonics frequencies are below 90 Hz, we set *N* = 7 here and the frequency of the *n*-th sub-band range from *n*× 8 Hz to 88 Hz. **W_X_**(**X**_**SBn**_,**Y**_**fk**_) and **W_Y_**(**X**_**SBn**_,**Y**_**fk**_) are the weight vectors consist of the first pair (maximum) of canonical variables in the CCA between **X**_**SBn**_ and **Y**_**fk**_. The better empirical values of *a*, *b*, and *N*_*h*_ are 1.25, 0.25, and 5 (Chen et al., 2015). And the frequency of **Y**_**fk**_ corresponding to the maximum ${\tilde{\rho }}_{k}$ is denoted as the stimulus frequency. Finally, the results of classification are transformed into commands and sent to the wheelchair or the simulated training scenario through the TCP/IP protocol.

### Simulated Training Scenario

**FIGURE 4:**
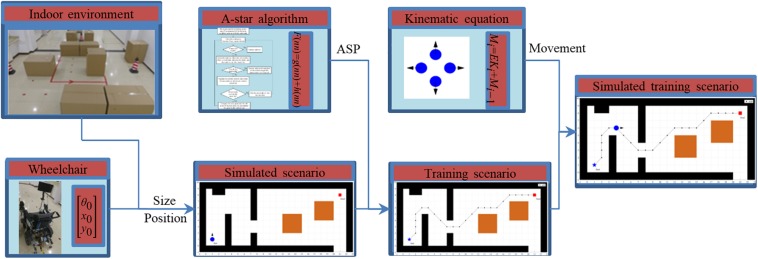
Diagram of the simulated training scenario.

A common room with about 13.2 m length and 5.4 m width is the prototype of the simulated scenario. The simulated training scenario provides an operator interface to set up different situations. Therefore, the obstacles and SSVEP-based BCW can be placed in the room or scenario according to the demand for training. Figure 4 illustrates the diagram of the simulated training scenario.

The space of the simulated scenario is divided into a grid map. According to the width (0.55 m) and length (1.10 m) of the wheelchair, the side length of the square grid corresponds to 0.6 m and the BCW is able to occupy about two grids. The grids are defined as obstacle grids and not allowed to pass while the obstacles belong to the grids. And the rest are defined as freedom grids. In this way, the coordinates of nodes are obtained from the vertices of respective grids. The position of obstacles is acquired by the web cameras fixed on the walls. The dimension information is deemed to the known quantity and input to the training scenario using a rectangular coordinate. The start point, the goal point, and the obstacle placement are determined in accordance with the training. In step-by-step BCW control, four parameters (the moving distance of the move-forward and move-backward, the rotation angle of the turn-right and turn-left) are able to be set according to the different situations. The simulated kinematical equation of BCW is given by:

(5) $${\text{M}}_{i}={\text{EK}}_{i}+{\text{M}}_{i-1}=\left[\begin{array}{c} \begin{array}{c} \theta_{i}\\ x_{i} \end{array}\\ y_{i} \end{array}\right]=\left[\begin{array}{ccc} d & 0 & 0\\ 0 & l & 0\\ 0 & 0 & l \end{array}\right]\,\left[\begin{array}{c} \begin{array}{c} \theta_{r}\\ \text{cos}\,\theta_{i} \end{array}\\ \text{sin}\,\theta_{i} \end{array}\right]+\left[\begin{array}{c} \begin{array}{c} \theta_{i-1}\\ x_{i-1} \end{array}\\ y_{i-1} \end{array}\right]$$

where ***M**_i_* is the motion coordinates vector; *i* represents the *i*-th step operation (*i* = 1, 2,…). ***E*** is the individual matrix determined by different situations (e.g., turning with motion or not); ***K**_i_* is the kinematic vector. *θ*_*i*_ is the heading angle (initial value is set to *θ*_*0*_); *θ*_*r*_ is the rotation angle and determined as a positive value when turning left (*d* = 1) and a negative value when turning right (*d* = −1). (*x*_*i*_, *y*_*i*_) is the position coordinate of BCW, and the initial value (*x*_0_, *y*_0_) is the position of the start point. *l* is the variable quantity of the moving distance.

### Path Planning

**FIGURE 5:**
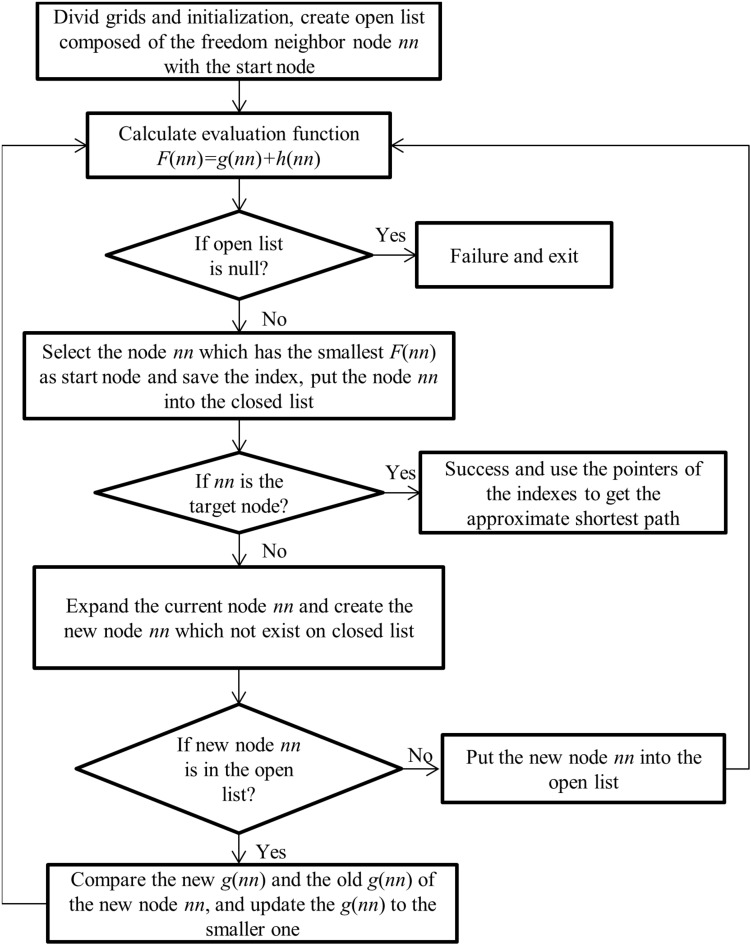
Flowchart of the acquisition of approximate shortest path with A-star algorithm.

In view of the operating space and accuracy, the A-star algorithm is used to search ASP. For security, the shortest safety distance between BCW and obstacles is determined to be equal to the half diagonal length of the grids (about 0.42 m in a real environment). We set the central initial position of BCW as the start point, and the neighbor freedom nodes are added to the queue list. The cost evaluation *F* of the path is calculated as following (Koenig and Likhachev, 2005):

(6) F⁢(n⁢n)=g⁢(n⁢n)+h⁢(n⁢n)

where *nn* is the next node within the neighbor freedom nodes on the path, *g*(*nn*) is the distance between *nn* and the start node on the path, *h*(*nn*), depends on the heuristic information, is the estimate of the distance between *nn* and the goal node. For the start point, all the *F* values of neighbor nodes are calculated. The next node *nn* is obtained from the node with the minimum value of *F*. In the same way, the node *nn* is replaced with its next node with the minimum value of *F* at each iteration, and the corresponding queue lists are updated as well. The loop iteration is stopped as the value of *h* is equal to zero. The approximate shortest path (ASP) is the line connected with the nodes which have the minimum value of *F*. The flowchart of the A-star algorithm is as shown in Figure 5.

For the convenience of design and realization, all programs are developed using the MATLAB environment.

### Experiment

#### Participants

To test the feasibility and performance, 10 healthy subjects (7 males and 3 females, 24 ± 3 years old and with normal or corrected-to-normal vision) who had never participated in any experiment on SSVEP-based BCW were recruited in the online training experiment. All of them signed the consent forms before the experiment. The experiment was performed according to the standards of the Declaration of Helsinki, and the study was approved by the Research Ethics Committee of Chinese Academy of Medical Sciences, China.

**FIGURE 6:**
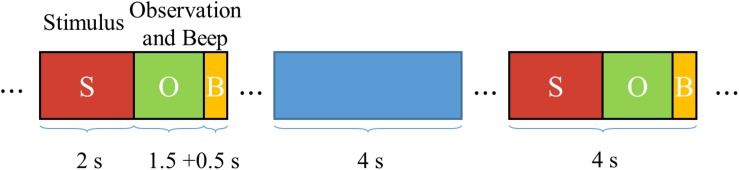
Temporal sequences diagram of the stimuli.

The paradigm of SSVEP stimuli of all tasks are exactly the same; each trial includes 2 s stimulus, 1.5 s observation, and 0.5 s beep, as shown in Figure 6. During the 2 s stimulus time, the subjects need to focus their attention on one of the stimulus targets. After the end of stimulus, subjects are asked to shift their gaze to the interface of simulated training scenario as soon as possible to observe the “road conditions” and determine the next state in 1.5 s. A 0.5 s beep sounding before the next trial is used to prompt the user to shift their gaze back to the stimulus targets.

#### Procedure

A total of four sessions for each subject in the training experiment are carried out, there are about 48 h rest between each session. And the integrated session consists of 5 (or 4) different tasks and one questionnaire survey. After each task, subjects are asked to take a 3 min break. Task 1 (T1) is designed to test the individual accuracy of subjects. In Task 2 (T2), Task 3 (T3), and Task 4 (T4), the level of difficulties increases gradually and subjects are asked to steer the simulated BCW from the start point to the goal point along ASP. In Task 5 (T5), ASP does not provide and the subjects should arrive at the goal point as safely and quickly as possible. In Session 1 (S1) and Session 3 (S3), subjects are requested to participate in T1 to T4 (a total of 10 tasks). T1 is carried out firstly (once), and then T2, T3, and T4 (training three times, respectively). In Session 2 (S2) and Session 4 (S4), subjects are requested to participate in T1 to T5 and the T5 is also performed three times (a total of 13 tasks). Finally, the questionnaire is filled out at the end of each session.

**FIGURE 7:**
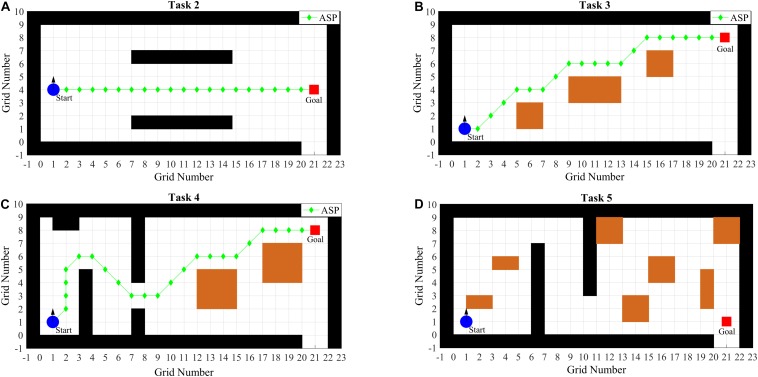
Different scenarios of T2 **(A)**, T3 **(B)**, T4 **(C)**, and T5 **(D)**.

To facilitate the operation, the rotation angle of turn-right or turn-left is set to π/4 and the moving distance of move-forward or move-backward is set to the half side length or half diagonal length (when the heading angle is an odd multiple of π/4) of the grid using Formula (5). The different scenarios of T2, T3, T4, and T5 are as shown in Figure 7. The specific descriptions of tasks are as follows:

T1: Individual accuracyTo evaluate the accuracy of the individual and the designed system, the random intentions (commands) task is carried out. A red triangle under a stimulus target is used as the cue of “gaze” during each trial and subjects should focus their attention on the cued targets. Each target is randomly repeated 20 trials (a total of 100 trials). The training scenario is set to a square with the 40 grids length of the side and has no obstacle.
T2: Simple linear pathThe subjects are asked to go through the corridor along ASP in this task. The simple linear path task without any obstacles is able to help the subjects to familiar with the simulated environment and practice moving in a straight line.
T3: Obstacles avoidanceThree obstacles are placed and the subjects are asked to turn left, right or right-angle to avoid them along ASP.

T4: Comprehensive scenarioThe scenario includes right-angle bend, S-shaped bend, corridor, door, and obstacles. ASP is provided as well.
T5: Evaluation taskS1 and S2 are the first stage of training; S3 and S4 are the second stage. T5 is designed to evaluate the training effect at the end of each stage. The difficulty of T5 is increased, and ASP is not provided. The subjects should determine the control paths by themselves in light of experiences obtained from training.

#### Scoring and Questionnaire

A score is designed to evaluate the performance and judge the task is successful or not. The time and distance to complete the task are closely related to the number of commands in step-by-step BCW movement, so the improvement before and after training can be clearly seen from the number of commands. At the same time, it is easy and simple to get information about safety in the driving process through the number of collisions. Therefore, the performance of the subjects in the tasks is mainly measured by the number of commands and collisions. Thereinto, the number of operation commands according to ASP is adopted as a reference, indicating the best performance that the subjects can achieve. The subjects should avoid collisions as much as possible. The full score is set to 100. To encourage participants, the task is deemed to fail as the result is less than or equal to 0. It does not matter whether a single task is successful or not, the whole task or session should be completed unless subjects give up voluntarily. The score is set as:

(7) $$S\,c=100-\frac{N_{a}-N_{\text{ASP}}}{N_{\text{ASP}}}\times 100-C$$

where *Sc* is the score of the subjects in the tasks. *N*_*a*_ represents the total number of actual operation commands, and *N*_ASP_ is the number of operation commands according to ASP. *C* is the number of collisions.

After each session, the questionnaire is asked to answer and the questions (Q1-Q4) are as following:

1. Can you stay concentrated in the whole session?
2. Do you think the tasks are difficult?
3. Do you feel tired in the tasks?
4. Do you feel uncomfortable about the stimuli?

The alternative answers are No (N), A little (A), Medium (M), Quite (Q).

### Performance Evaluation

To evaluate the improvement of subjects after training with the simulated training environment, the results of T1 to T4 in S1 and T5 in S2 are set as the control group and compared with those of other sessions. In addition to using paired-samples *t*-test to compare the results, one-way repeated-measures analysis of variance (ANOVA) is also applied to determine the statistical significance of differences in the accuracy.

## Results

In T1, there is no obstacle in the scenario, and subjects need to gaze the stimulus following the cues. The accuracies including the average values (AVG) and standard deviations (STD) are listed in Table 1. It can be found that the maximum is up to 100.00%, and the grand average of accuracies is 93.55%. Thereinto, the average accuracies of S1 to S4 are increases gradually and the result of S4 is 96.50 ± 3.95%. A paired-sample *t*-test reveals an improvement on the accuracy. And the average accuracy improves from 91.05% in the first stage (S1 and S2) to 96.05% in the second stage (S3 and S4) (*p* = 0.001). Comparing S1 and S4, the average accuracy increases from 90.30% in the S1 to 96.50% in the S4 (*p* < 0.05). In addition, one-way repeated-measures ANOVA is also applied to analyze the differences of accuracy in Table 1. The results show that there is a statistically significant difference between these four sessions [*F*(3, 36) = 3.50, *p* < 0.05]. Pairwise comparisons reveal a significant difference between S1 and S4 (*p* < 0.05). And there is no significant difference between S1 and S2 (*p* > 0.05), no significant difference between S1 and S3 (*p* > 0.05). All of these results are considered to be acceptable.

**TABLE 1:** The accuracy of subjects.

**Subjects**	**Accuracy (%)**			
	**S1**	**S2**	**S3**	**S4**
Sub1	96.00	94.00	98.00	98.00
Sub2	79.00	93.00	97.00	98.00
Sub3	88.00	77.00	93.00	87.00
Sub4	88.00	96.00	100.00	100.00
Sub5	100.00	96.00	100.00	100.00
Sub6	89.00	92.00	89.00	96.00
Sub7	83.00	91.00	97.00	100.00
Sub8	95.00	92.00	91.00	97.00
Sub9	93.00	90.00	97.00	94.00
Sub10	92.00	97.00	94.00	95.00
AVG ± STD	90.30 ± 6.25	91.80 ± 5.69	95.60 ± 3.72	96.50 ± 3.95

**FIGURE 8:**
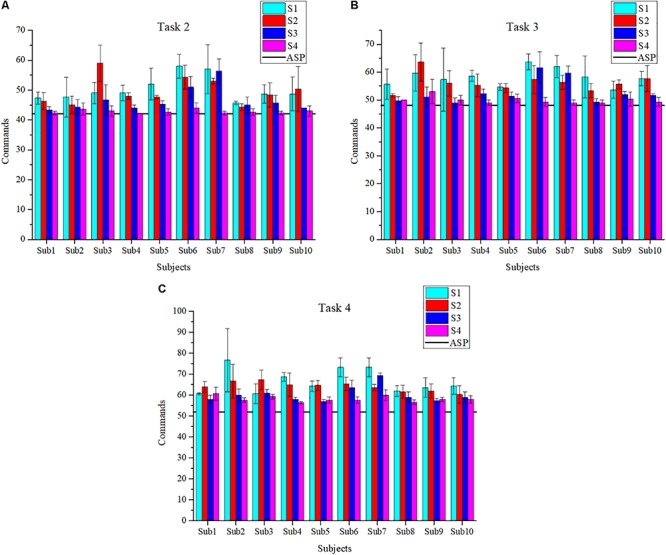
The average number of commands in T2 **(A)**, T3 **(B)**, and T4 **(C)**.

In T2, T3, and T4, ASPs are provided, which can help subjects practice turning right-angle, S-shaped bend, crossing the corridor and door, and obstacles avoidance. The scores and the number of commands are used to assess the performance of subjects as far as possible. And the number of commands of ASP is used as a reference, which are 42, 48, and 52 in T2, T3, and T4, respectively. To facilitate the observation of results, the histograms of the average number of commands in each task are plotted in Figure 8. The collisions and average scores of each session in the experiment are also calculated and as shown in Table 2.

**TABLE 2:** The collisions and average scores in T2, T3, and T4.

**Subjects**	**Tasks**	**Scores (AVG ± STD)/Collisions**			
		**S1**	**S2**	**S3**	**S4**
Sub1	T2	87.30 ± 4.96/0	89.68 ± 6.88/0	96.83 ± 2.75/0	99.21 ± 1.37/0
	T3	84.03 ± 11.47/0	92.36 ± 1.20/1	96.53 ± 3.18/0	95.83 ± 0.00/0
	T4	91.33 ± 1.41/1	85.72 ± 4.72/0	96.43 ± 3.57/0	91.67 ± 5.46/0
Sub2	T2	86.51 ± 15.85/0	92.86 ± 7.15/0	94.45 ± 5.99/0	96.03 ± 4.95/0
	T3	75.69 ± 13.55/0	67.03 ± 14.36/1	93.75 ± 7.51/0	89.58 ± 9.08/0
	T4	62.76 ± 27.64/1	80.62 ± 14.89/1	92.52 ± 5.86/1	97.02 ± 2.06/0
Sub3	T2	83.33 ± 8.59/0	59.53 ± 41.48/0	88.22 ± 13.05/2	97.62 ± 4.12/0
	T3	79.89 ± 24.81/2	83.00 ± 9.12/1	97.92 ± 3.61/0	95.83 ± 3.61/0
	T4	91.67 ± 8.25/0	79.75 ± 8.43/0	90.74 ± 3.67/1	94.05 ± 2.06/0
Sub4	T2	83.33 ± 6.30/0	85.71 ± 2.38/0	95.24 ± 2.38/0	100.00 ± 0.00/0
	T3	77.78 ± 4.34/0	84.72 ± 8.42/0	90.97 ± 3.18/0	97.92 ± 2.08/0
	T4	77.38 ± 3.72/0	83.93 ± 9.94/0	96.43 ± 1.79/0	99.40 ± 1.03/0
Sub5	T2	76.19 ± 12.60/0	86.51 ± 1.38/0	92.06 ± 2.75/0	98.41 ± 2.75/0
	T3	86.11 ± 2.41/0	86.81 ± 3.18/0	93.06 ± 2.41/0	94.44 ± 3.18/0
	T4	84.79 ± 5.03/1	84.52 ± 4.12/0	98.21 ± 1.79/0	97.02 ± 2.72/0
Sub6	T2	61.90 ± 9.52/0	90.63 ± 9.62/0	77.90 ± 9.70/2	95.24 ± 4.12/0
	T3	67.36 ± 6.01/0	79.89 ± 11.43/2	70.53 ± 11.85/3	97.22 ± 3.18/0
	T4	69.05 ± 3.72/0	83.33 ± 5.74/0	85.98 ± 6.75/1	97.02 ± 2.73/0
Sub7	T2	64.29 ± 19.49/0	73.81 ± 2.38/0	65.87 ± 19.10/0	99.21 ± 1.37/0
	T3	70.50 ± 8.84/1	82.64 ± 5.24/0	75.69 ± 5.24/0	97.92 ± 2.08/0
	T4	69.05 ± 8.05/0	85.64 ± 1.68/1	75.86 ± 2.40/1	92.86 ± 4.72/0
Sub8	T2	91.27 ± 1.37/0	94.44 ± 2.75/0	92.86 ± 6.30/0	98.41 ± 2.75/0
	T3	78.47 ± 15.64/0	88.89 ± 5.24/0	97.22 ± 2.41/0	97.92 ± 2.08/0
	T4	89.29 ± 4.72/0	89.88 ± 5.46/0	94.31 ± 5.29/1	98.48 ± 2.64/1
Sub9	T2	84.13 ± 7.27/0	84.92 ± 9.62/0	91.27 ± 5.99/0	99.21 ± 1.37/0
	T3	88.19 ± 6.36/0	83.69 ± 2.64/1	91.67 ± 2.08/0	95.14 ± 5.24/0
	T4	86.31 ± 8.44/0	89.29 ± 6.19/0	97.62 ± 2.06/0	95.76 ± 2.84/2
Sub10	T2	84.13 ± 13.54/0	79.83 ± 17.82/1	95.24 ± 0.00/0	97.62 ± 4.12/0
	T3	79.53 ± 5.72/2	79.53 ± 9.35/0	92.36 ± 1.20/0	97.22 ± 3.18/0
	T4	84.79 ± 7.32/1	92.26 ± 7.43/0	94.64 ± 4.72/0	96.43 ± 3.09/0

In T2, the scenario is relatively simple and subjects are asked to control the BCW go through the corridor along ASP. It can be seen from Figure 8A that subjects perform better and better except Sub3 in S2, because Sub3 makes some mistakes and costs lots of commands for corrections in that session. The average number of 59 commands is executed, which is much greater than 42 commands of ASP. But during S4, the average number of 43 commands is cost, which is very close to 42. T3 is mainly constructed to practice turning and obstacles avoidance. As can be seen from Figure 8B, the overall performance of each subject tends to be better from S1 to S4. In addition, the results of S3 and S4 (second stage) are better than those of S1 and S2 (first stage). Figure 8C shows the average number of commands in T4. With the exception of Sub1 and Sub3, the average number of commands used by each subject to complete the tasks is gradually reduced. The most impressive results belong to Sub2, and the average number of commands is 76.67, 66.67, 60.00, and 57.67 from S1 to S4.

In addition, the paired-sample *t*-test is adopted to analyze the difference of all subjects between S1 and S4. The average and standard deviation of commands are 58.40 ± 9.14 and 50.32 ± 6.56, respectively. The average number of commands decreases by 8.08 and the standard deviation decreases by 2.58, and is significantly different (*p* < 0.001). Note that the average number of commands in the synthesis of T2, T3, and T4 according to ASP is only 48.67. It indicates that the operational proficiency of subjects improves through the training with ASP.

According to the scores in Table 2, it can be found that the performance of subjects is improved between S1 and S4. The T2 of Sub7 shows the most significant improvement, the average score increases by 34.92 (from 64.29 ± 19.49 of S1 to 99.21 ± 1.37 of S4). Meanwhile, the paired-sample *t*-test is used to analyze the difference in the scores of S1 and S4 of all subjects. The average score of S4 (96.66 ± 3.70) is 16.78 higher than that of S1 (79.88 ± 12.64), and the difference is significant (*p* < 0.001). And the standard deviation decreases by 8.94. These results further show that the performance has improved and become more stable after the training. Regarding the total number of collisions in each task, there are five collisions in T2, 13 collisions in T3 which mainly occurred in S1 and S2, and 14 collisions in T4 since the scenario is slightly difficult and comprehensive.

**FIGURE 9:**
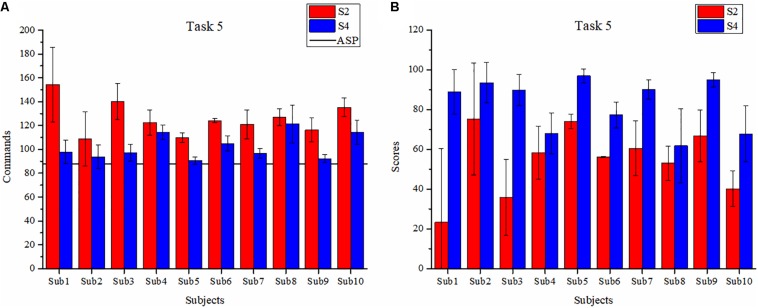
The average number of commands **(A)** and scores **(B)** in T5.

The main aim of T5 is to assess the training effect. The first T5 is scheduled at the end of S2, and the latter is scheduled in S4. The number of commands and collisions of T5 in S2 and S4 are as shown in Table 3, the average number of commands and scores are as shown in Figure 9.

**TABLE 3:** The number of commands and collisions in T5.

	**S2**			**S4**		
**Subjects**	**Commands/Collisions**			**Commands/Collisions**		
Sub1	181/4	162/0	120/0	109/0	93/0	91/0
Sub2	135/4	95/0	96/0	104/0	89/0	88/0
Sub3	126/3	138/5	156/7	105/0	94/0	92/0
Sub4	134/4	113/2	121/1	113/0	121/6	109/0
Sub5	114/0	110/1	106/2	94/0	88/0	90/0
Sub6	122/5	125/2	125/2	112/1	99/3	104/4
Sub7	112/4	116/0	135/2	98/0	100/0	92/0
Sub8	119/2	132/4	130/2	139/1	117/0	108/0
Sub9	107/0	127/3	115/0	96/0	91/0	90/0
Sub10	143/7	136/3	126/8	126/5	110/2	107/0

Regarding Table 3, the mean number of collisions is 2.57 in S2, since the scenario is harder than that of T4 and ASP is not provided for subjects. The average number of commands is 125.90, which is much higher than the number of commands in ASP (88). In S4, the performance of subjects has improved. The average number of commands is 102.30 with the number of collisions dropping to 0.73. In terms of the average number of commands, the result of S4 (102.30 ± 12.53) is 23.60 less than that of S2 (125.90 ± 18.35) and the difference is significant (*p* < 0.001) for paired-sample *t*-test. Especially, the average number of commands of the last T5 is 97.1, which is close to the commands of ASP.

As can be seen from Figure 9A, the average number of commands for all subjects shows a downward trend as a whole. In S4, the number of commands used by all subjects to complete T5 is close to the number of commands of ASP (88). According to the scores shown in Figure 9B, the average scores of S4 are higher than those of S2. In S4, all the subjects could complete T5 with better performance, and the maximum is 96.97 ± 3.47. The average score of S4 (82.95 ± 15.13) in T5 is 28.30 higher than that of S2 (54.65 ± 21.14), the difference being significant (*p* < 0.001) for paired-sample *t*-test. And the standard deviation decreases by 6.01. These results verify that all subjects achieve an improved performance of BCW control.

**FIGURE 10:**
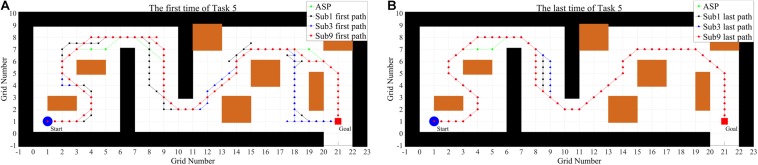
The specific paths of three subjects in the first T5 of S2 **(A)** and the last T5 of S4 **(B)**.

For T5, Figure 10 shows the first and the last paths of Sub1, Sub3, and Sub9, who earn the lowest, medium and highest scores in the first test, respectively. It shows the specific paths of subjects. It can be found that the subjects steer basically according to previous experience, and the actual paths are similar to ASP, especially the last paths.

The comprehensive results of the questionnaire are shown in Table 4. For each question, we calculate the average score of all subjects’ answers in each session and mark the alternative answer closest to the average score as a comprehensive result. Here, N, A, M, and Q correspond to scores of 25, 50, 75, and 100, respectively. As seen in Table 4, the subjects are able to stay concentrated and do not feel very uncomfortable in the whole experiment. The subjects feel a little fatigue in all sessions, which satisfies the mental workload required for training. According to the evaluation of the difficulty, the tasks are acceptable, although subjects feel a little difficult at the beginning of the experiment. It indicates that the simulated training environment is available to the subjects.

**TABLE 4:** The comprehensive results of the questionnaire.

**Questions**	**S1**	**S2**	**S3**	**S4**
Q1. Concentration	Q	M	Q	Q
Q2. Difficulty	A	N	N	N
Q3. Fatigue	A	A	A	A
Q4. Discomfort	N	A	N	N

*N: No, A: A little, M: Medium, and Q: Quite.*

## Discussion

Improving the proficiency and efficiency of BCW control is very important for users. The main aim of our study is to design and apply an indoor simulated training environment to train and improve users’ performance in using SSVEP-based BCW which is evaluated by classification accuracy and scoring (or commands). The system can help users to understand the indoor situation, which users may encounter in daily life, by establishing a simulated environment from an overhead perspective. The control proficiency of users will be improved through repeated training in different situations, such as going straight, turning left or right, turning right-angle and S-shaped bend, crossing the corridor and door, and obstacles avoidance, etc. Furthermore, the complexity of training is gradually increased to improve the mental workload of users. So, training in this indoor simulated environment is beneficial to the performance improvement of users and help them learn and become proficient in BCW control.

In a similar approach, some simulated systems or neurofeedback training systems for BCI have been developed in the recent past. Related work has described introducing a hybrid BCI that uses the MI-based mu rhythm and the P300 potential to control a brain-actuated simulated or real wheelchair (Long et al., 2012). All of the subjects accomplished predefined tasks successfully and obtained an average accuracy of 83.10%. In the other study, SSVEP-based BCI has succeeded in the control of a brain-controlled simulated vehicle (Bi et al., 2014). Four participants were required to perform a driving task online to test the system. The average accuracy is 76.87% and the mean ratio of task completion time to the nominal time (i.e., time optimality ratio) is 1.36. In more recent work, a simulated driving system based on hybrid BCI and computer vision was proposed to explore and verify the feasibility of human-vehicle collaborative driving (Li et al., 2018). The information transfer rate (ITR) in the on-line experiment reaches 85.80 bits/min and the task success rate is 91.1%. Besides, a recently published paper designed a virtual automatic car based on multiple patterns of MI-based BCI (Wang et al., 2019). The participants were asked to navigate the virtual automatic car through the six predefined destinations sequentially. The experimental results showed that the average accuracy is 75% and the mean time optimality ratio reaches 1.28.

In comparison with the abovementioned simulated systems (Long et al., 2012; Bi et al., 2014; Li et al., 2018; Wang et al., 2019), which aimed to verify and test the feasibility and performance of the designed BCW or BCI systems, the present study designed and applied an indoor simulated training environment to improve the performance of users in BCW control. As results of the individual accuracy task, the average accuracy is 93.55% and the maximum individual accuracy is 100.00% (see Table 1). In terms of time optimality ratio, we get the results by calculating the ratio of the actual number of commands in tasks to the nominal number of commands in ASP (4 s per command). And the mean ratio of T2, T3, and T4 is 1.15 while the result is 1.30 in T5. These results substantiate that all subjects are able to use the SSVEP-based BCI to issue commands with high accuracy. As for ITR, according to the formula of Yuan et al. (2013), the average ITR is 28.26 ± 4.65 bits/min and the maximum is 34.83 bits/min (with 5 targets and 4 s of each trial). And ITR will decrease as the time of each trial increases, however, the users need more time to observe the situation since the obstacles are usually close to the BCW in the indoor environment.

In recent years, training has been used to improve the performance of some BCI systems. Relevant to MI-based BCIs, Kus et al. (2012) designed an asynchronous BCI based on MI and improved performance through neurofeedback training. After training, the participants used the asynchronous MI-based BCI to navigate the cursor through the maze and achieved a mean ITR of 4.51 bits/min and a mean accuracy of 74.84%. Recently, Yao et al. (2019) proposed a sensory stimulation training approach to improve the performance of a BCI based on somatosensory attentional orientation (Yao et al., 2019). Results showed that a significantly improved accuracy of 9.4% has been realized between the pre- and post-training and the average accuracy after training is 78.6%. In the aspect of P300-based BCIs, a study found that training can improve tactile P300-based BCI performance within a virtual wheelchair navigation task (Herweg et al., 2016). And mean accuracy improved from 88.43% in the 1st to 92.56% in the last session while the ITR increased from 4.5 bits/min to 4.98 bits/min. Similarly, another study also investigated the effect of training on performance of BCI with an auditory P300 multi-class speller paradigm (Baykara et al., 2016). Subjects were asked to spell several words by attending to animal sounds representing the coordinates of the letter in the matrix. The ITR increased from 3.72 bits/min to 5.63 bits/min after five training sessions. The previous study also demonstrated that alpha neurofeedback training improves SSVEP-based BCI performance (Wan et al., 2016). The training group showed an average increase of 16.5% in the SSVEP signal SNR (signal-to-noise ratio) and the average accuracy improved from 65.4% to 78.7%. Unlike the above training systems (Kus et al., 2012; Baykara et al., 2016; Herweg et al., 2016; Wan et al., 2016; Yao et al., 2019), the main purpose of this study is to achieve performance improvements in BCW control trough the training environment. However, it is worthwhile mentioning that the average accuracy is improved and the mean ITR is also increased (*p* < 0.001). Although the improved accuracy and ITR are better than the results of the above studies (Kus et al., 2012; Baykara et al., 2016; Herweg et al., 2016; Wan et al., 2016; Yao et al., 2019), we pay more attention to the improvement of users’ control ability and performance. With regard to control performance, the number of commands decreases significantly while the score is significantly increased (*p* < 0.001). All of these compared results prove that the performance of subjects in BCW control is improved and verify the feasibility of the proposed environment.

Different from the previous simulated BCW systems (Leeb et al., 2007; Gentiletti et al., 2009; Huang et al., 2012; Francisco et al., 2013; Herweg et al., 2016), the training scenario in this work is modeled according to the real indoor environment (e.g., home, hospital), and the position, size of the objects are almost the same. The work (operation steps and process) required the subjects to control BCW from the starting point to the goal point in the simulated environment is basically the same as that in the real environment. Users will be familiar with the environment in advance while training. Meanwhile, the ASP obtained by path planning can be used to assist the driving route selection in a real environment. And the training scenario also provides an operator interface to adjust parameters (e.g., size, shape, and obstacle placement) according to the actual situation. Users are able to choose some routes commonly used in daily life for specific training. For the simulated wheelchairs, the motion parameters (e.g., speed, rotation angle) can be set and adjusted to meet the different real motion. Furthermore, the simulated training environment is not limited to SSVEP-based BCW. In order to satisfy the needs of practical applications better, this paper chooses to use SSVEP-based BCI to get user commands and as a control input for the environment. According to the characteristics of ERD/ERS-based BCI and P300-based BCI, better training effects are predictable.

On the other hand, there are aspects in the following that need to be further improved. The evaluation matrix can be further improved by adding more factors (e.g., fatigue) and whether the users can carry out the BCW control in the real environment can be assessed by scoring in the future. Secondly, the SSVEP-based BCI adopted a synchronous protocol, which cannot discriminate the control and idle states of subjects. Adding an ON/OFF switch is one of the good solutions for this problem (Cheng et al., 2002). Finally, the simulated scenario is designed into a 2D space for the convenience of path planning and recommendation. The virtual reality (VR) technology can be used to build a more high-quality simulated environment (Rutkowski, 2016). In addition, although only 10 subjects conducted four training sessions within 7 days, the feasibility and training effect of the environment has been proved with the satisfactory results of subjects. Next, we will conduct simulated training of ERD/ERS-based BCW or P300-based BCW to further verify the performance of the environment, and invite more subjects (or disabled subjects) to participate in the experiment.

## Conclusion

In this study, an indoor simulated training environment for SSVEP-based BCW control training is built. The 7-days training experiment includes individual accuracy task and other four tasks with or without ASP are designed and validated. The cases of turning right-angle and S-shaped bend, crossing the corridor and door, and obstacles avoidance are practiced. Scoring and command-consuming are considered to measure the performance and training effect of the subjects. The results of experiments show that the subjects can realize the control training of BCW through our indoor simulated training environment and achieve a certain degree of improvement, and prove the feasibility and effectiveness of the environment.

## Data Availability Statement

The datasets and programs generated for this study are available on request to the corresponding author.

## Ethics Statement

The studies involving human participants were reviewed and approved by the Research Ethics Committee of Chinese Academy of Medical Sciences, China. The patients/participants provided their written informed consent to participate in this study.

## Author Contributions

ML and KW designed the research. ML performed the research. XC, KW, and ML wrote the programs. ML, YC, and JZ analyzed the data. ML, KW, and JZ wrote the manuscript. HW, JW, and SX provided facilities and equipment.

## Conflict of Interest

The authors declare that the research was conducted in the absence of any commercial or financial relationships that could be construed as a potential conflict of interest.
